# MXene Coatings: Novel Hydrogen Permeation Barriers for Pipe Steels

**DOI:** 10.3390/nano11102737

**Published:** 2021-10-16

**Authors:** Kejun Shi, Xinyu Meng, Shu Xiao, Guohua Chen, Hao Wu, Chilou Zhou, Saihua Jiang, Paul K. Chu

**Affiliations:** 1 Institute of Safety Science & Engineering, South China University of Technology, Guangzhou 510641, China; 18796287706@163.com (K.S.); xinyum2021@126.com (X.M.); mmghchen@scut.edu.cn (G.C.); wuhao@scut.edu.cn (H.W.); mezcl@scut.edu.cn (C.Z.); 2 Department of Physics, City University of Hong Kong, Tat Chee Avenue, Kowloon, Hong Kong 999077, China; paul.chu@cityu.edu.hk; 3 Department of Materials Science and Engineering, City University of Hong Kong, Tat Chee Avenue, Kowloon, Hong Kong 999077, China; 4 Department of Biomedical Engineering, City University of Hong Kong, Tat Chee Avenue, Kowloon, Hong Kong 999077, China

**Keywords:** MXenes, hydrogen barrier, hydrogen embrittlement, corrosion resistance, coatings

## Abstract

MXenes are a new class of two-dimensional (2D) materials with promising applications in many fields because of their layered structure and unique performance. In particular, the physical barrier properties of two-dimensional nanosheets make them suitable as barriers against hydrogen. Herein, MXene coatings were prepared on pipe steel by a simple spin-coating process with a colloidal suspension. The hydrogen resistance was evaluated by electrochemical hydrogen permeation tests and slow strain rate tests, and the corrosion resistance was assessed by potentiodynamic polarization. The results reveal that MXene coatings offer excellent hydrogen resistance and corrosion protection by forming a barrier against diffusion. Experimentally, the hydrogen permeability of the MXene coating is one third of the substrate, and the diffusion coefficient decreases as well. The mechanistic study indicates that the hydrogen resistance of the MXene coatings is affected by the number of spin-coated layers, while the concentration of the d-MXene colloidal suspension determines the thickness of a single coating. However, damage to the sample surface caused by the colloidal suspension that contains H^+^ and F^−^ may limit the improvement of the hydrogen resistance. This paper reveals a new application of 2D MXene materials as a novel efficient barrier against hydrogen permeation and the subsequent alleviation of hydrogen embrittlement in the steel substrate.

## 1. Introduction

Hydrogen energy is important to modern society due to its natural abundance, large energy density, recyclability, and environmental friendliness. However, hydrogen embrittlement is one of the reasons for the reduced lifetime of components used in the transmission and containment of hydrogen such as pipes, and mitigation of the damage introduced by hydrogen embrittlement is crucial to the hydrogen energy storage and transportation industries [1,2,3]. The pipes used to transport hydrogen are generally made of metallic materials which are typically prone to hydrogen embrittlement that entails plastic loss and accelerated fatigue cracking. In addition, current hydrogen transportation methods mostly use natural gas pipelines and blend hydrogen with natural gas. This leads to many corrosion problems. In order to improve the safety of hydrogen transportation, hydrogen barrier coatings have attracted research interest in the hydrogen energy industry [1].

Although different types of hydrogen barrier coatings such as oxide [4,5,6], silicon carbide [7], and iron aluminum alloys [8] have been reported, the application of conventional hydrogen barrier coatings is plagued by delamination and cracking, poor thermal stability, and strict production conditions [3]. Two-dimensional (2D) materials have been shown to offer good hydrogen resistance and corrosion protection [9]. For example, the hydrogen permeability of graphene coatings deposited on copper is about 28 times smaller than that of pristine copper. If it is normalized to the thickness, the graphene–hydrogen barrier actually shows a larger permeation reduction factor than most conventional hydrogen barrier coatings [10]. Hexagonal boron nitride films deposited on yttrium-doped zirconia by atomic layer deposition improve not only the hydrogen resistance but also thermal stability [11]. Recently, a new class of graphene-like nanomaterials called MXenes has aroused interest in the fields of energy storage, catalysis, tribology, and membrane separation technology [12,13]. Spin coating is commonly employed to prepare 2D MXene coatings due to the precise control of the film thickness, easy operation, and the almost pollution-free nature of the process [14]. In fact, the molecular sieve [15,16,17] and hydrogen storage [18] characteristics of the MXene make it a potential hydrogen barrier coating, but the relevant hydrogen barrier properties have not been studied systematically so far.

In this study, the Ti_n+1_C_n_T_x_ 2D MXene coatings were deposited on the X70 pipe steel by spin coating, and the hydrogen barrier properties were evaluated by electrochemical hydrogen permeation tests. Two-dimensional MXene coatings prepared with different concentrations of the ingredients in the suspensions were prepared for comparison and characterized by scanning electron microscopy (SEM), X-ray diffraction (XRD), and Fourier transform infrared spectroscopy (FT-IR). The corrosion resistance and tensile properties were also assessed to gauge the commercial viability of the hydrogen barrier coatings and elucidate the associated hydrogen resistance mechanism.

## 2. Experimental Section

### 2.1. Materials

The Ti_3_AlC_2_ (99 wt%, ~400 mesh) was purchased from Laizhou Kai Kai Ceramic Materials Co., Ltd. (Laizhou, China), and the lithium fluoride (LiF, AR) and hydrochloric acid (HCl, AR) were obtained from Guangzhou Qianhui Chemical Glass Instrument Co., Ltd. (Guangzhou, China). All the chemicals were used as received without purification.

### 2.2. Synthesis of the d-MXene Flake Solution

The MXene (Ti_3_C_2_T_x_) nanosheets were synthesized by etching with HCl and LiF [19]. Firstly, 2 g of LiF were dissolved in 40 mL of 9 mol/L HCl solution to form the etching solution. Two grams of Ti_3_AlC_2_ were added slowly into the etching solution under magnetic stirring at 45 °C for 24 h to dissolve the Al. The resulting suspension containing the multilayered Ti_3_C_2_T_x_ was rinsed several times with deionized (DI) water, centrifuged at 3500 rpm (5 min for each cycle), and decanted until the pH of the dark green supernatant was 6. The Ti_3_C_2_T_x_ was mixed with absolute ethanol, sonicated in an ice bath for 1 h, and centrifuged for 10 min at 10,000 rpm to obtain the sediment. The DI water was then added to the sediment and sonicated for 20 min to promote delamination of the Ti_3_C_2_T_x_ flakes. Finally, the colloidal suspension with the d-MXene nanosheets was obtained after centrifugation for 5 min at 3500 rpm.

### 2.3. Fabrication of MXene Coatings on Pipe Steel

The X70 pipe steel substrate was cut into 50 mm × 30 mm × 1 mm rectangular pieces by wire cutting, polished with sandpaper to a mirror finish, cleaned with acetone and ethanol ultrasonically, and dried. The substrate was then coated using the prepared d-MXene colloidal suspensions with different concentrations (4 mg/mL, 2 mg/mL, 1 mg/mL, and 0.5 mg/mL). Spin coating proceeded in two steps: at 500 rpm (30 s) and at 2000 rpm (10 s).

### 2.4. Electrochemical Hydrogen Permeation Evaluation

To study the hydrogen permeation resistance of the MXene coatings, hydrogen permeation tests were performed with the aid of the Devanathan-Stachurski (D-S) cell [20] in which aqueous saturated calomel electrode (SCE) was the reference electrode, platinum foil was the counter electrode, and 0.2 mol/L NaOH was the electrolyte. Before the hydrogen permeation test was performed, a layer of nickel was plated on the uncoated side of the sample. In order to reduce the background current to below 0.1 μA/cm^2^, a constant potential of 200 mV (vs. SCE) was applied to the anodic cell, and 3 g/L CH_4_N_2_S was added to the cathode cell to prevent the hydrogen from escaping. Finally, a constant current of −12 mA/cm^2^ was applied to the cathode to start the test. The apparent diffusion coefficient *D*_app_ and hydrogen permeability *J* were calculated by Equation (1):

(1) $$D_{\mathrm{app}}=\frac{L^{2}}{15.3t_{b}}$$

where *t*_b_(s) is the breakthrough time representing when the hydrogen permeation current starts to rise and *L*(cm) is the thickness of the specimen. According to the current density *I*_t=40,000s_, the hydrogen permeability *J* of the specimen was calculated by Equation (2):

(2) $$J=\frac{I_{t=40,000s}\times L}{F}$$

where *F* is Faraday’s constant with a value of 96,485 C/mol.

### 2.5. Slow Strain Rate Measurement

The slow strain rate test (SSRT) was used to assess the mechanical properties of the samples before and after the hydrogen charging to gauge the resistance against hydrogen embrittlement (HE). The non-standard specimen (15 mm long in the parallel section, 4 mm wide in the gauge length section, and 1 mm in thickness) was used for the test. The test of the uncoated and coated samples, which were pre-charged with hydrogen at a current density of 50 mA/cm^2^ for 24 h using the two-electrode system, was carried out as soon as possible after charging to avoid thermal desorption and loss of hydrogen. In the experiment, the strain rate was 5.5 × 10^−5^/s.

### 2.6. Electrochemical Characterization

The corrosion behavior of the coated and uncoated specimens was evaluated at room temperature using a conventional three-electrode electrochemical cell consisting of a platinum mesh as the counter electrode (CE), a saturated calomel electrode (SCE) as the reference electrode, a coated or uncoated specimen with an exposed area of 1 cm^2^ as the working electrode, and 0.1 mol/L NaCl as the electrolyte. Potentiodynamic polarization was carried out in the voltage range from −900 mV to −300 mV vs. OCP at a constant scanning rate of 1 mV/s.

### 2.7. Materials Characterization

The surface morphology and elemental distributions were analyzed using a scanning electron microscope (SEM, Merlin, Zeiss, Oberkochen, Germany) equipped with an energy-dispersive X-ray spectrometer (EDS). An optical microscope (KWONG KUK, Shenzhen, China) was utilized to observe the corroded surface, and X-ray diffraction (XRD, Smartlab, Rigaku, Tokyo, Japan) was performed to determine the structure of the MAX and synthesized MXene powders. Fourier transform infrared (FTIR) spectroscopy was conducted on the Thermo Scientific Nicolet iS5 FTIR in the range of 400–4000 cm^−1^ to analyze the functional groups in the MXene powder.

## 3. Results and Discussion

### 3.1. Fabrication and Characterization

**Figure 1:**
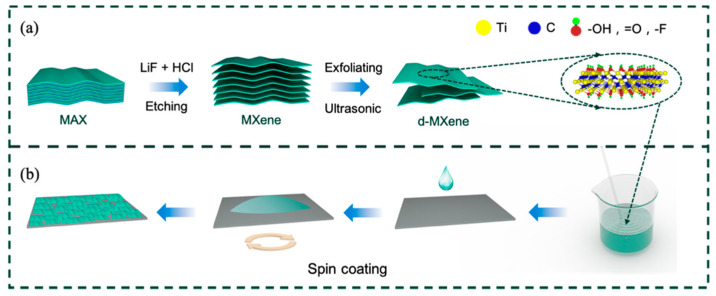
Schematic illustration of the fabrication process of (**a**) MXene nanosheets and (**b**) MXene coating.

**Figure 2:**
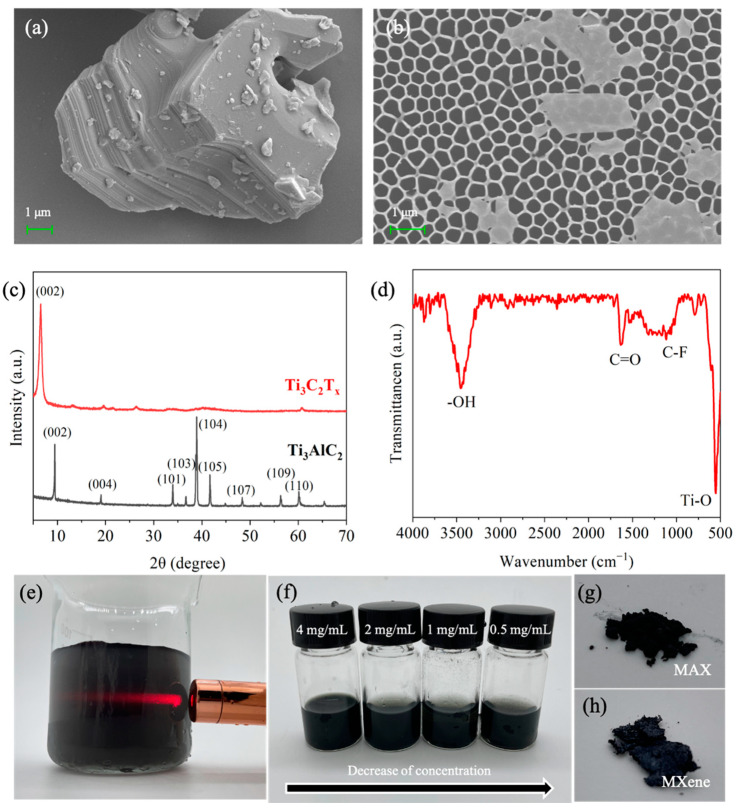
SEM images of (**a**) Ti_3_AlC_2_ and (**b**) d-Ti_3_C_2_T_x_; (**c**) XRD patterns of Ti_3_AlC_2_ and d-Ti_3_C_2_T_x_; (**d**) FTIR spectrum of d-Ti_3_C_2_T_x_; (**e**) Optical photograph of d-Ti_3_C_2_T_x_ in DI water; (**f**) Samples with different concentrations of d-Ti_3_C_2_T_x_; (**g**,**h**) Photographs of the powders.

As shown in Figure 1, the fabrication process of the MXene coatings on the X70 steel substrate includes two key steps. In the first step (Figure 1a), the colloidal suspension containing the d-MXene nanosheets is prepared by selective etching the Al interlayer from the MAX followed by exfoliation into ultrathin flakes. Figure 2a depicts the typical layered structure showing micromechanical cleavage of the bulk MAX before etching. After the first step, Figure 2b reveals that mono- or few-layer freestanding 2D MXene flakes are exfoliated from the multi-layered Ti_3_C_2_T_x_ assisted by sonication, indicating that the intercalation of Li^+^ between the Ti_3_C_2_T_x_ layers is effective for delamination. The XRD patterns in Figure 2c corroborate the formation of the MXene. After etching, the characteristic (104) peak of the Ti_3_AlC_2_ at 39° weakens, and the (002) diffraction peak corresponding to the 2D Ti_3_C_2_T_x_ shifts to a smaller angle, indicating that the Al atoms in the MAX are fully removed, and the interlayer spacing increases [21]. The FTIR spectra (Figure 2d) show peaks at 554 cm^−1^, 1100 cm^−1^, 1632 cm^−1^, and 3445 cm^−1^ associated with the stretching vibrations of the Ti-O, C-F, C=O, and -OH bonds in accordance with previous reports [22]. Figure 2e presents the Tyndall scattering effect of the colloidal suspension of the d-MXene; the colloidal state is crucial for the formation of the coatings by spin coating. As shown in Figure 2f, the colloidal suspension is diluted to different concentrations, and the color becomes lighter with reduced concentration. The optical photos of the MAX and MXene powders show small differences in color, and the larger interlayer spacing after etching increases the volume (Figure 2g,h).

**Figure 3:**
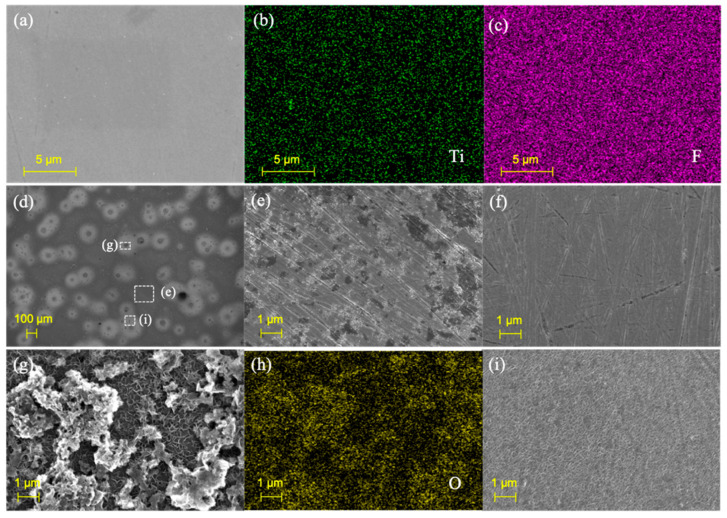
(**a**,**d**) Morphology; (**b**,**c**) Element distributions of the MXene coatings deposited on pipe steel; (**e**,**g**,**i**) Surface SEM images of the local area in (**d**); (**f**) Surface SEM image of the steel substrate; (**h**) EDS image of (**g**).

**Figure 4:**
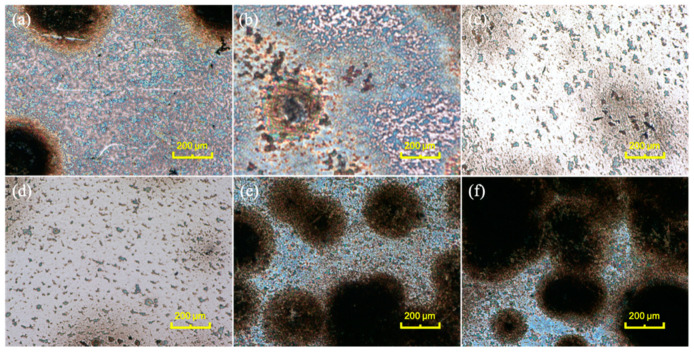
Surface optical microscopy images of the MXene coatings fabricated under different conditions: (**a**) 4 mg/mL, 1 layer; (**b**) 2 mg/mL, 1 layer; (**c**) 1 mg/mL, 1 layer; (**d**) 0.5 mg/mL, 1 layer; (**e**) 4 mg/mL, 2 layers; (**f**) 4 mg/mL, 3 layers.

In the second step (Figure 1b), the MXene coatings are prepared on the steel substrate with the d-MXene colloidal suspension by spin coating. According to the surface morphology and elemental distributions shown in Figure 3a–c, Ti and F are distributed uniformly in the MXene coatings. Figure 3d depicts the morphology of the steel surface after spin coating, and three different morphologies are observed, namely local area (e), area (g) and area (i). Figure 3e shows the primary morphology coated with MXene flakes in area (e), which is obvious in comparison with the substrate (Figure 3f). As shown in Figure 3g,h, the dark region in area (g) is oxide, indicating that the agglomerated MXene particles are oxidized after exposure to air. Figure 3i shows the micrograph of area (i) revealing that a small part of the surface is corroded by H^+^ and F^−^ from the colloidal suspension. Optical microscopy is used to investigate the effects of different colloidal suspension concentrations and spin-coated layers on the corrosion resistance. As shown in Figure 4a–d, when the concentrations of H^+^ and F^−^ decrease, the area and degree of corrosion decrease. By comparison, in Figure 4a,e,f, surface corrosion intensifies with an increasing number of spin-coated layers because each spin-coating step adds new corrosion areas and expands them.

### 3.2. Hydrogen Permeation Behavior of the MXene Coatings

**Figure 5:**
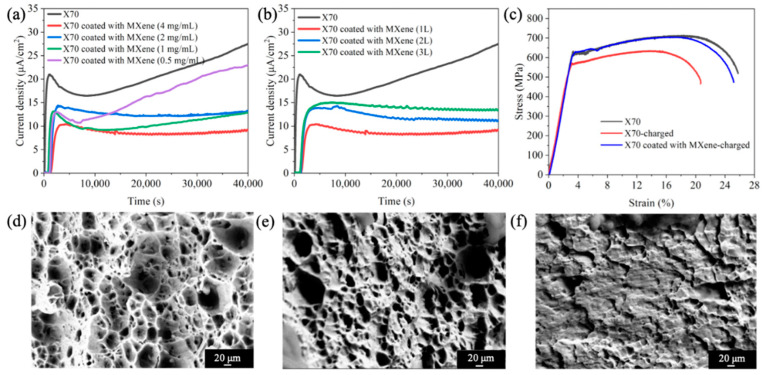
(**a**,**b**) Electrochemical hydrogen permeation curves; (**c**) Strain–stress curves of the uncoated and coated samples (MXene-4 mg/mL, 1 L) after electrochemical hydrogen charging; Fracture morphologies of (**d**) X70 without hydrogen charging, (**e**) X70 with hydrogen charging, and (**f**) MXene-4 mg/mL-1 L with hydrogen charging.

Figure 5a–b displays the hydrogen permeation curves of the uncoated and coated X70 steel samples, and the permeation parameters derived from the hydrogen permeation curves are listed in Table 1. Figure 5 shows that the bare X70 steel is easily permeated by the hydrogen, as manifested by a large *I*_t=40,000s_ (27.5 μA·cm^−1^) and a short *t*_b_ (110 s). Moreover, *D* and *J* of the uncoated sample are calculated to be 5.94 × 10^−6^ cm^2^·s^−1^ and 2.85 × 10^−5^ mol·cm^−1^·s^−1^ by Equations (1) and (2), respectively. After the X70 steel substrate is coated with the MXene (4 mg/mL, 1 L), smaller *I*_t=40,000s_ and longer *t*_b_ are observed. *D* of the MXene (4 mg/mL, 1 L) coated sample is 4.91 × 10^−7^ cm^2^·s^−1^, and *J* decreases by more than two thirds. By reducing the MXene concentration or increasing the spin-coated layer number, both *D* and *J* decrease, indicating poorer resistance against hydrogen permeation.

SSRT is applied to characterize the HE in steels by quantifying the loss of ductility due to the influence of diffused hydrogen induced by electrochemical hydrogen charging. Figure 5c exhibits the strain–stress curves of the uncoated and coated samples (MXene-4 mg/mL-1 L) after electrochemical hydrogen charging. A typically good plasticity is observed in the uncharged X70 steel sample. The tensile behavior of the charged X70 steel is different from that of the uncharged sample, indicating the occurrence of HE. When the X70 steel samples are covered by MXene coatings, the strain-stress curve is similar to that of the uncharged bare sample even though the coated sample has been charged for a long time. Therefore, the MXene coatings improve HE resistance in the X70 steel. The corresponding fracture morphologies of the uncoated and coated samples charged after SSRT are shown in Figure 5d–f. In the absence of hydrogen, the X70 steel shows the typical dimple morphology, and the large dimple size suggests good plasticity. After charging for a long time, dissociative fracture characteristics reflecting hydrogen-induced fractures are observed on the uncoated X70 steel samples, revealing the transformation from plastic fractures to brittle fractures. On the other hand, after charging the MXene-coated X70 steel samples, the fracture morphology retains the dimple pattern as well as the plastic fractures similar to the uncharged sample.

### 3.3. Corrosion Resistance

**Figure 6:**
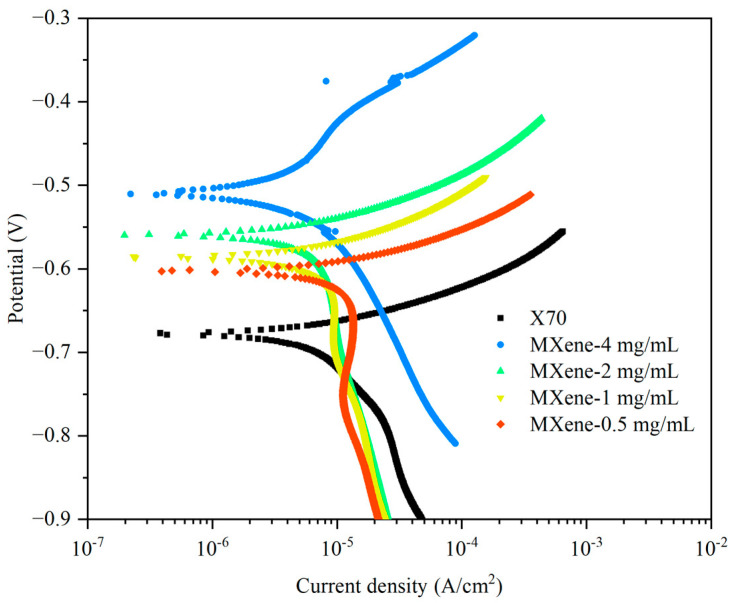
Tafel curves of the substrate and samples with different concentrations.

Potentiodynamic polarization is utilized to analyze the corrosion characteristics of the MXene coatings as shown in Figure 6, and Table 2 summarizes the electrochemical properties, including the corrosion potential *E*_corr_ and the corrosion current density *I*_corr_. The uncoated X70 steel shows the *E*_corr_ = −0.681 V and the *I*_corr_ = 5.6 μA·cm^−2^. After coating with MXene (4 mg/mL), a positive shift of about 170 mV is observed, implying that the MXene is less susceptible to corrosion after immersion for 30 min. Meanwhile, the *I*_corr_ decreases to 1.5 μA·cm^−2^ suggesting that the coatings provide protection to the substrate underneath. However, both the *E*_corr_ and the *I*_corr_ decrease with decreasing MXene concentration. The decrease in the andic current density, which is a measure of the metal dissolution and corrosion rate, suggests that the larger defect density in the MXene coatings reduces corrosion resistance.

### 3.4. Mechanism

**Figure 7:**
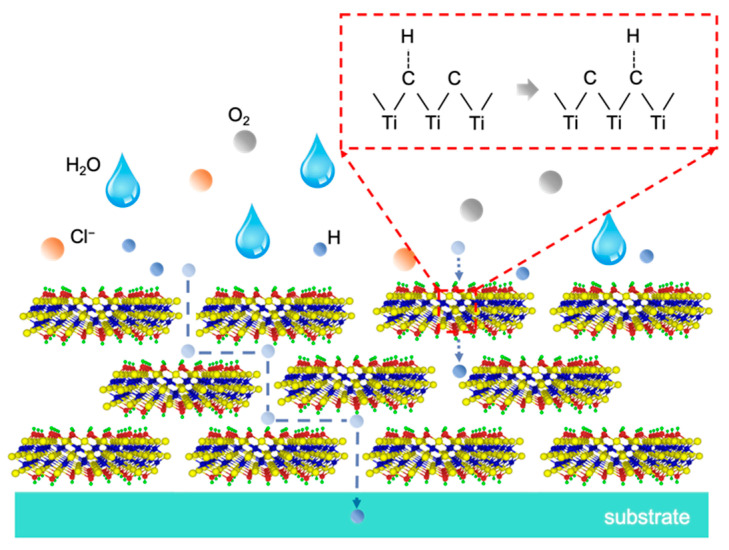
Schematic presentation of hydrogen permeation and corrosion processes.

It is evident that MXene inhibits hydrogen permeation and corrosion based on two factors (Figure 7). Firstly, the physical barrier effects are endowed by the multilayered MXene. After spin coating, the d-MXene is stacked to form a two-dimensional, multi-layered structure as a physical barrier. Although the interlayer space in the MXene coatings becomes the preferred diffusion channels for hydrogen, the tortuous diffusion paths effectively prolong the diffusion time. Hence, the MXene coatings mitigate the diffusion of the electrolyte and provide excellent corrosion protection [23]. Secondly, the interactions between hydrogen and MXene produce desirable effects. Ceramic coatings block hydrogen molecules because ions are formed in the process of hydrogen permeation [24,25,26,27]. Hydrogen atoms entering the coating break the bonds near the coating surface and combine with the host atoms to form ions. The ions reorganize in the coating and break bonds around it. The hydrogen atoms then break away from the original bonds and recombine with new elements to form ions. In this process, hydrogen atoms continue to break bonds until they reach the metal substrate. Some researchers have suggested that a large number of CH_4_^−^ ions are formed in TiC films to resist hydrogen diffusion [28,29,30]. Therefore, the decreased hydrogen permeability observed in the MXene coatings is mainly because enough energy is needed to break the Ti-C bonds and form C-H bonds when hydrogen atoms diffuse. Based on this barrier mechanism, the hydrogen resistance of the MXene coatings is affected by the number of layers, while the concentration of the d-MXene colloidal suspension determines the thickness of a single spin coating. When the concentration decreases, the number of MXene layers also decreases, resulting in enhanced permeability. Theoretically, increasing the spin coating time increases the coating thickness and improves the hydrogen resistance. However, damage to the sample caused by the colloidal suspension which contains H^+^ and F^−^ becomes more severe and may degrade the hydrogen resistance.

## 4. Conclusions

MXene coatings are prepared on the X70 pipe steel by simple spin coating with a colloidal suspension, and the hydrogen resistance and corrosion resistance of the coatings are evaluated systematically. The MXene (Ti_3_C_2_T_x_) nanosheets prepared by the spin coating and etching have the desirable 2D single-layer structure. The MXene coatings improve the hydrogen resistance, and the permeability *J* of the MXene coating (4 mg/mL-1 L) is one third of that of the uncoated steel substrate. The diffusion coefficient *D* decreases as well. The MXene coating provides an anticorrosion physical barrier as manifested by the *E*_corr_ showing a positive shift of 170 mV compared to the uncoated steel as well as a smaller *I*_corr_. Increasing the number of spin coatings also increases the coating thickness and improves the hydrogen resistance. However, damage to the sample caused by the colloidal suspension, which contains H^+^ and F^-^ becomes more severe and may degrade the hydrogen resistance. All in all, 2D MXene coatings with improved hydrogen resistance reduce the risk of hydrogen embrittlement in commercial steels and have tremendous commercial potential.

## Figures and Tables

**Table 1:** Permeation parameters derived from the hydrogen permeation curves.

Samples	Breakthrough Time *t*_b_/s	Diffusion Coefficient *D*/cm^2^·s^−1^	Permeation Current *I*_t=40,000s_/μA·cm^−1^	Permeability *J*/mol·cm^−1^·s^−1^
X70	110	5.94 × 10^−6^	27.5	2.85 × 10^−5^
MXene-4 mg/mL-1 L	1330	4.91 × 10^−7^	9.1	9.43 × 10^−6^
MXene-2 mg/mL-1 L	1200	5.45 × 10^−7^	13.1	1.36 × 10^−5^
MXene-1 mg/mL-1 L	850	7.69 × 10^−7^	12.9	1.34 × 10^−5^
MXene-0.5 mg/mL-1 L	820	7.97 × 10^−7^	23.0	2.38 × 10^−5^
MXene-4 mg/mL-2 L	1290	5.07 × 10^−7^	11.3	1.17 × 10^−5^
MXene-4 mg/mL-3 L	1120	5.84 × 10^−7^	13.5	1.40 × 10^−5^

**Table 2:** Electrochemical properties of the coatings derived from the Tafel curves.

Samples	*E*_corr_ (V vs. SCE)	*I*_corr_ (μA·cm^−2^)
X70	−0.681	5.6
MXene-4 mg/mL	−0.511	1.5
MXene-2 mg/mL	−0.561	3.3
MXene-1 mg/mL	−0.588	4.2
MXene-0.5 mg/mL	−0.605	5.6

## Data Availability

The data presented in this study are available on request from the corresponding author.
